# Supplementary figures and images for: Lactate receptor HCAR1 regulates neurogenesis and microglia activation after neonatal hypoxia-ischemia
Source: eLife. 2022 Aug 9;11:e76451. doi: 10.7554/eLife.76451 (PMC9363115; doi:10.7554/eLife.76451)

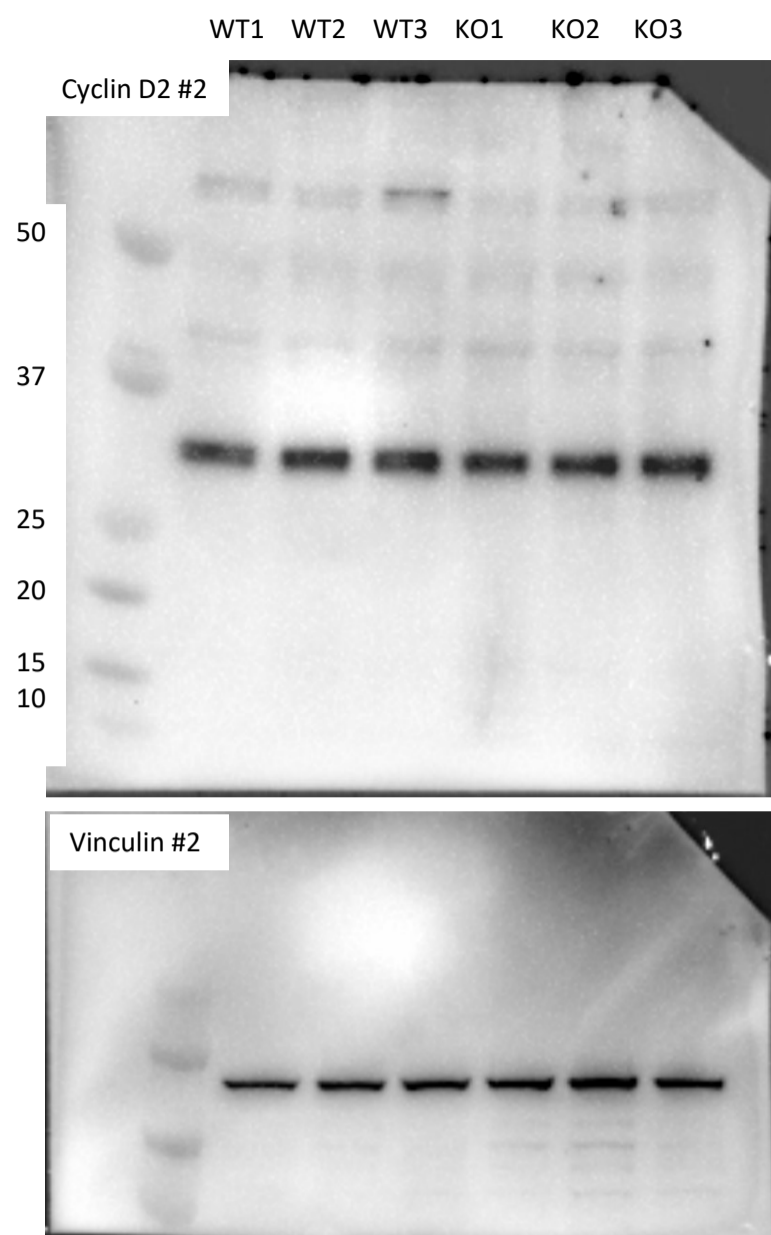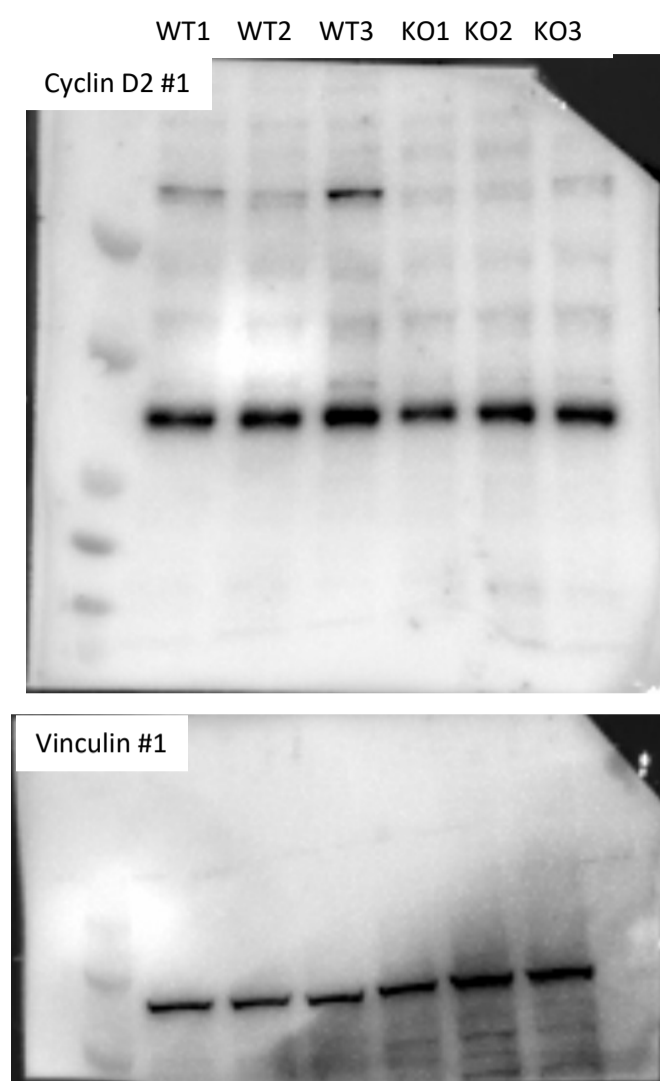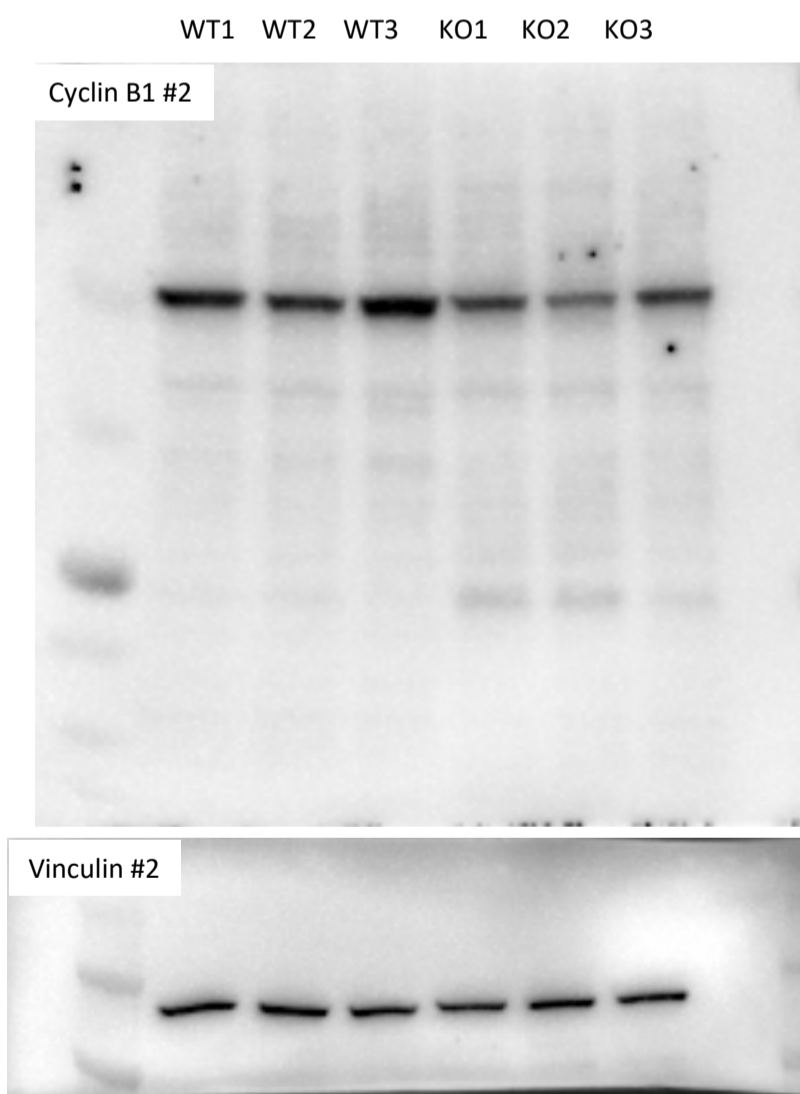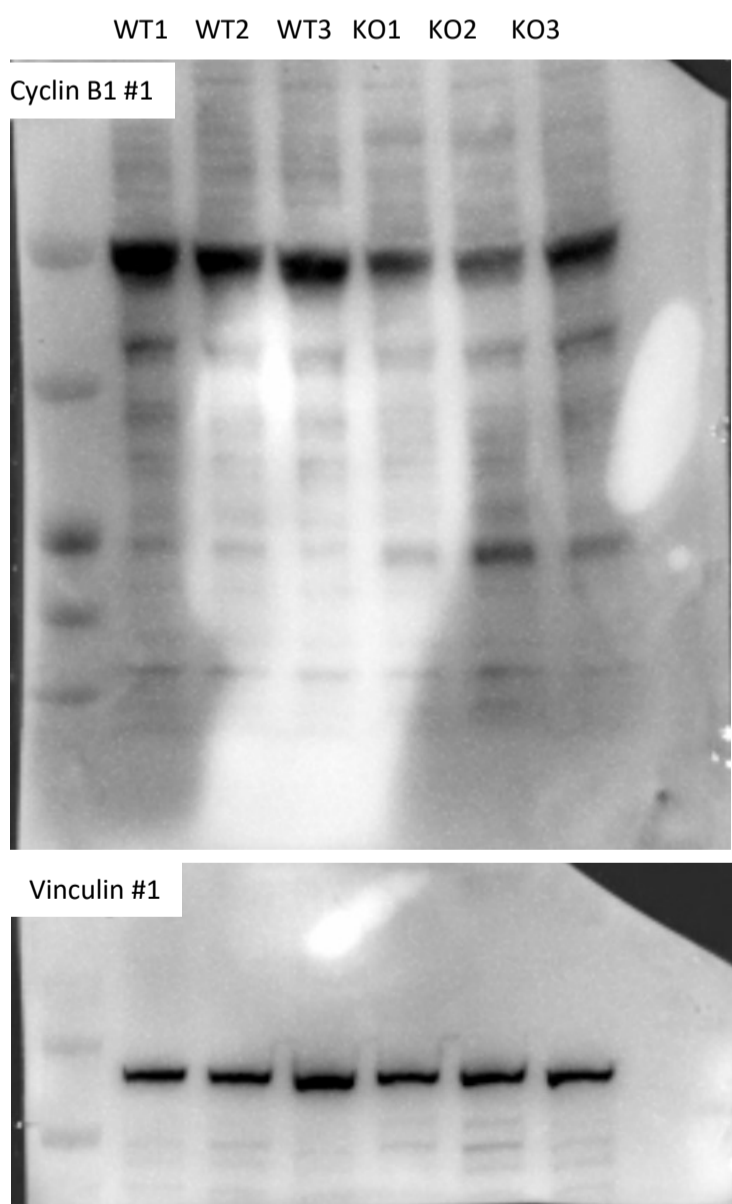

Supplement: Figure 5—source data 1. [file elife-76451-fig5-data1.zip › Western Blot PDF raw images.pdf]
