# Supplementary material for: Lactate receptor HCAR1 regulates neurogenesis and microglia activation after neonatal hypoxia-ischemia
Source: eLife. 2022 Aug 9;11:e76451. doi: 10.7554/eLife.76451 (PMC9363115; doi:10.7554/eLife.76451)
Supplement: Supplementary file 1. — Thresholds ->logFC ≥ 0.5 (UP DEGs) and ≤–0.5 (DOWN DEGs); all of them significant at adjusted P-value <0.05. SVZ, subventricular zone; hc, hippocampus. [file elife-76451-supp1.docx]

| **Supplementary file 1. Number of differentially expressed genes (DEGs) between the different experimental groups** | | | |
| --- | --- | --- | --- |
| **Comparisons** | **Total UP DEGs** | **Total DOWN DEGs** | **Total DEGs** |
| HCAR1.svz.ipsi vs WT.svz.ipsi | 3258 | 3182 | 6440 |
| WT.svz.ipsi vs WT.svz.contra | 3594 | 3738 | 7332 |
| HCAR1.svz.contra vs WT.svz.contra | 8 | 3 | 11 |
| HCAR1.svz.ipsi vs HCAR1.svz.contra | 251 | 501 | 752 |
| HCAR1.hc.ipsi vs WT.hc.ipsi | 33 | 4 | 37 |
| WT.hc.ipsi vs WT.hc.contra | 2894 | 2298 | 5192 |
| HCAR1.hc.contra vs WT.hc.contra | 10 | 3 | 13 |
| HCAR1.hc.ipsi vs HCAR1.hc.contra | 4167 | 3625 | 7792 |
| *Thresholds -> logFC ≥ 0.5 (UP DEGs) and ≤ -0.5 (DOWN DEGs); all of them significant at adjusted p-value < 0.05. SVZ, subventricular zone; hc, hippocampus.* | | | |
